# Histologic evidence of neutrophil extracellular traps and fibrin(ogen) deposition in liver biopsies from patients with inflammatory liver disease

**DOI:** 10.1016/j.rpth.2024.102666

**Published:** 2024-12-31

**Authors:** Fien A. von Meijenfeldt, Ton Lisman, Alessandra Pacheco, Yoh Zen, William Bernal

**Affiliations:** 1 Surgical Research Laboratory, Department of Surgery, University Medical Center Groningen, Groningen, the Netherlands; 2 Institute of Liver Studies, King’s College Hospital, London, United Kingdom; 3 Liver Intensive Care Unit, Institute of Liver Studies, King’s College Hospital, London, United Kingdom

**Keywords:** cirrhosis, coagulation, liver injury, platelets, thrombosis

## Abstract

**Background:**

Liver disease is often characterized by the activation of coagulation and inflammation. Experimental studies suggest that the interaction between neutrophils and platelets with local activation of coagulation could contribute to liver injury progression, but there have been limited studies in humans.

**Objectives:**

We studied the hemostatic components and neutrophil extracellular traps (NETs) in liver biopsies from patients with different inflammatory liver diseases.

**Methods:**

Liver biopsies from patients with inflammatory liver disease (alcoholic steatohepatitis [ASH], autoimmune hepatitis, primary sclerosing cholangitis, metabolic-associated steatohepatitis, and allograft ischemia-reperfusion injury (IRI), each *n* = 20) were stained for fibrin(ogen), platelets, and NETs. The correlation of NET formation with deposition of hemostatic components and laboratory measures of disease severity was investigated.

**Results:**

In 75% of the liver biopsies, no fibrin(ogen) was detectable, and only 20% of the biopsies showed minimal deposition. Overall, 50% of liver biopsies stained positive for NETs. Platelet deposition and NET formation were highest in IRI, where it correlated with histologic severity of injury (r = .61 [95% CI, .22-.84]; *P* < .01) and ASH. Platelet deposition was associated with NET formation (r = .44 [95% CI, .27-.59]; *P* < .001) and colocalized in the biopsies. NET formation, but not fibrin and platelet deposition, was moderately associated with the model of end-stage liver disease score (r = .29 [95% CI, .07-.49]; *P* < .01).

**Conclusion:**

In contrast to experimental studies, we demonstrated minimal intrahepatic fibrin(ogen) deposition in different types of human inflammatory liver disease. Histologic evidence for intrahepatic NETs was common and most pronounced in acute ASH and IRI and was associated with platelet deposition and disease severity.

## Essentials

• Hemostasis and immunothrombosis contribute to liver disease progression in animal models.
• We studied hemostatic components in human liver biopsies to expand published preclinical data.
• Contrasting published animal studies, we found little hemostatic components in human livers.
• Our results highlight the difficulty of extrapolating results from animal studies into patients.

## 1 Introduction

Liver disease is characterized by marked changes in the hemostatic system that might contribute to its progression. Plasma levels of most hemostatic proteins decrease proportionally to the severity of the disease, but due to simultaneous changes in pro- and anticoagulant proteins, the hemostatic system often remains in balance, and both bleeding and thrombotic complications are rare in stable disease [1]. However, in some patients with liver disease, the hemostatic system has notable prothrombotic features [[2], [3], [4], [5]]. Experimental studies suggest that activation of the hemostatic system might contribute to the progression of liver disease [6,7].

Although there is evidence for the activation of hemostasis in humans with liver disease [8], clinical evidence of a role for activation of coagulation in the progression of liver disease in humans is primarily based on studies of patients with (congenital) hematologic disorders, such as factor (F)V Leiden [9], or studies using anticoagulant therapy that was shown to decrease disease progression [10,11]. In a randomized controlled trial on the use of anticoagulation in patients with cirrhosis to prevent portal vein thrombosis (PVT), anticoagulant treatment with low-molecular-weight heparin not only reduced the incidence of PVT but also that of acute decompensations and was associated with improved liver function and survival [10]. A more recent meta-analysis suggests that anticoagulation is associated with reduced mortality in patients with PVT, independent of recanalization of the portal vein [11]. These reports suggest that inhibiting the activation of coagulation could delay the progression of liver disease and that further mechanistic and clinical studies are warranted.

In multiple experimental studies using rodent models of liver fibrosis, acute liver injury, and liver failure, anticoagulation decreases liver injury, implying a functional role in the activation of coagulation in its progression [6,[12], [13], [14], [15]]. One proposed mechanism of injury is the formation of microthrombi, leading to local hepatic ischemia and injury. Experimental studies have demonstrated substantial amounts of intrahepatic fibrin(ogen) accumulating in the liver following various types of liver injury [6]. Prophylactic administration of anticoagulant therapy was shown to reduce both fibrin(ogen) deposition and liver injury or the development of fibrosis [6,16]. However, whether fibrin(ogen) drives liver disease is increasingly questioned, as studies using functional fibrin(ogen)-deficient mice did not find evidence of a difference in liver damage in a model of acetaminophen-induced liver injury [17,18]. Interestingly, intrahepatic fibrin(ogen) deposition contributed to liver repair after partial hepatectomy in mice and patients [19], implying a protective rather than detrimental role for fibrin(ogen). It might thus be that the role of fibrin(ogen) depends on the type of injury and disease, and any role in the progression of disease remains to be clarified.

Another proposed mechanism by which coagulation proteases could contribute to disease progression is through activation of hepatic stellate cells via protease-activated receptors (PARs). Hepatic stellate cells express PAR-1 and PAR-2. The former is primarily activated by thrombin, and inhibition of PAR-1 signaling in a number of models reduced liver fibrosis [16]. PAR-2, which can be activated by FXa or neutrophil elastase (NE), has also been implicated in liver fibrosis [20].

NE is an important component of neutrophil extracellular traps (NETs), structures released by activated neutrophils that can activate coagulation and promote thrombosis and are implicated in the pathogenesis of multiple diseases [21,22]. As neutrophils often accumulate in the injured liver, NETs could contribute to the progression of liver disease by activation of coagulation. In a mouse model of portal hypertension, the formation of NETs resulted in the formation of microthrombi that promoted the progression of portal hypertension and liver disease [23]. Further, in a mouse model of acute alcoholic hepatitis, NETs contributed to disease progression [24]. Clinically, elevated plasma markers of NETs in patients with acute liver failure were associated with death or urgent liver transplantation [25], suggesting a potential role of NETs in the progression of human acute liver injury. In a study of liver biopsies from patients with metabolic-associated steatohepatitis (MASH), NET deposition was demonstrated in the majority and was associated with histologic severity of disease scores [26].

Though the interplay between neutrophils, platelets, and local activation of coagulation has been proposed to contribute to the progression of liver injury, clinical data to support this suggestion are very limited. We, therefore, studied the deposition of fibrin(ogen), NETs, platelets, and the platelet adhesive protein von Willebrand factor (VWF) in liver biopsies from 100 patients with different types of liver disease with inflammatory phenotypes and their relation with the nature and severity of liver disease.

## 2 Methods

### 2.1 Study cohort

Liver biopsies obtained after informed consent from adult patients with liver disease (≥18 years) collected between 2019 and 2022 at King’s College Hospital, London, and stored in the diagnostic archive were used for this study. These comprised 20 patients with acute alcoholic steatohepatitis (ASH), 20 patients with MASH, 20 patients with primary sclerosing cholangitis (PSC), and 20 patients with autoimmune hepatitis (AIH). In addition, 20 biopsies from allograft livers after implantation during liver transplantation (postreperfusion biopsies) were included. Ethical approval for the study was granted by the Heath Research Authority (Research Ethics Committee Reference 22/PR/1740). All biopsies were anonymized before use and linked to relevant clinical and laboratory data in an anonymized database. Laboratory values were obtained on the day of the biopsy, if available, or approximately to the day of the biopsy.

### 2.2 Histologic grading and staging

Paraffin-embedded liver biopsies were sectioned and stained with hematoxylin and eosin, reticulin, or Sirius red. Histologic analyses were performed by an experienced liver pathologist (Y.Z.). In liver sections from patients with ASH, inflammatory activity and fibrosis stage were determined based on the criteria proposed by the Study of Alcohol-related LiVer disease in Europe (SALVE) [27]. In this system, the degree of steatosis, activity, canalicular cholestasis, ductular cholestasis, and degree of fibrosis are separately scored. Activity is based on the sum of hepatocellular injury and lobular neutrophil scores. In addition, in allograft livers, the degree of ischemia-reperfusion injury (IRI) was classified as minimal (only focal neutrophilic infiltrate or hepatocellular necrosis), mild (scattered foci of hepatocyte necrosis and neutrophilic aggregates), moderate (perivenular confluent necrosis), or severe (bridging or pan-lobular necrosis).

### 2.3 Immunohistochemistry

Immunohistochemical stainings were performed using an automated staining machine (Bond-Max autostainer, Leica). The following antibodies were used: citrullinated histone H3 (H3cit; Abcam, ab5103) to quantify NETs, CD61 (Leica, PA0308) to quantify platelets, and fibrin(ogen) (Abcam, Ab58207). In cases of ASH and postreperfusion allograft biopsies, we stained liver sections for VWF (Dako, A0082) and NE (Bio-Connect, NP57), a marker for neutrophils. More detailed information on immunohistochemical approaches is provided in Supplementary Table S1.

**Figure 1:**
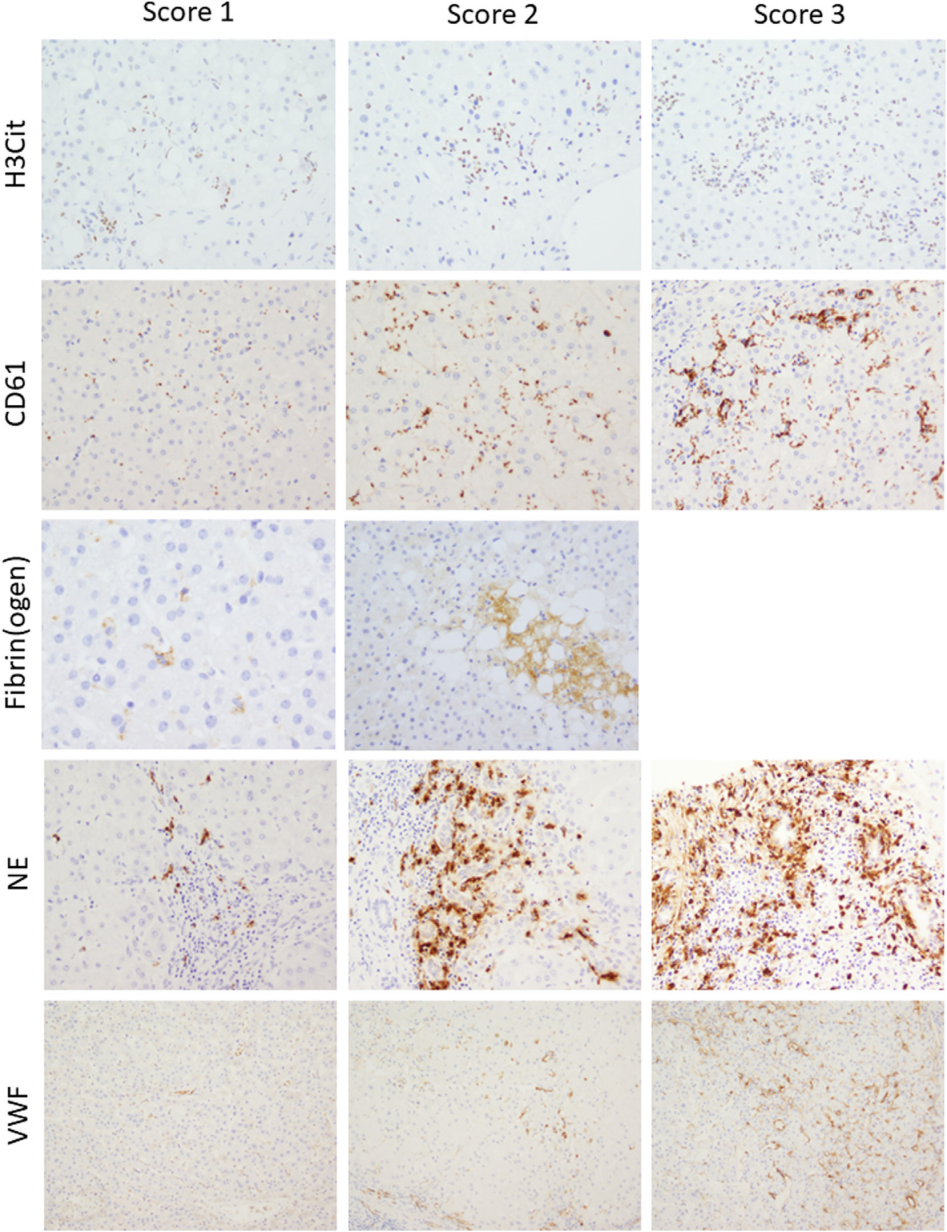
Representative pictures of immunohistochemical stainings of human liver tissue. Examples of semiquantitative scoring systems 1 to 3 for neutrophils and hemostatic components. For fibrinogen, only scores 1 and 2 were included, as none of the biopsies showed a score of 3. Citrullinated histone H3 (H3Cit) was used as a marker for neutrophil extracellular traps, CD61 as a marker for platelets, and neutrophil elastase (NE) as a marker for neutrophils. VWF, von Willebrand factor.

Immunohistochemical results were semiquantitatively examined. The histologic appearance of cells was taken into account with the quantification. Representative pictures of the semiquantitative scoring system are shown in Figure 1. A representative higher magnification picture of CD61 platelet staining showing small granular positive staining inside sinusoids is shown in the Supplementary Figure.

### 2.4 Statistical analysis

Patient characteristics are reported as median with IQR for continuous variables and numbers and percentages for categorical variables. Differences between groups were calculated using the Mann–Whitney U-test or one-way analysis of variance, as appropriate. The relation between the expression of different markers and clinical characteristics was analyzed using Spearman’s correlation test and shown as correlation r with 95% confidence interval. Statistical significance was defined as *P* < .05. All analyses were performed with the use of GraphPad Prism version 9.3.1.

## 3 Results

### 3.1 Patient characteristics

A total of 100 patients were included in this study. Table 1 shows the characteristics of the study population. Patients with PSC were younger than those with MASH and liver transplant donors. Compared with the other groups, patients with ASH had significantly higher model of end-stage liver disease (MELD) scores [28], total bilirubin levels, and international normalized ratios (INRs), and lower platelet counts and albumin levels. Twelve (60%) of the patients with acute ASH were inpatients in the intensive care unit at the time of biopsy compared with 1 in the MASH and none in the AIH and PSC groups. In contrast, 15 (75%) of the PSC patients underwent the liver biopsy as an outpatient. Aspartate aminotransferase (AST) levels in the postreperfusion group were significantly higher than in the other groups, and platelet counts were lower in blood tests taken from the liver transplant recipients shortly after transplantation.

**Table 1:** Patient characteristics.

Variable	IRI^a^ (*n* = 20)	ASH (*n* = 20)	MASH (*n* = 19)^b^	PSC (*n* = 20)	AIH (*n* = 20)	*P* value
**Female (%)**	6 (30)	14 (70)	13 (65)	7 (35)	12 (60)	
**Age, y**	56 (44-61)	46 (33-53)	58 (47-62)	34 (24-50)	53 (33-62)	<.01
**Location** Outpatient/ward/ICU (no. of patients)		0/8/12	9/9/1	15/5/0	8/12/0	
**MELD score** (original)	n/a	24 (21-28)	6 (6-6)	6 (6-10)	9 (7-17)	<.0001
**Laboratory results**						
Albumin (g/L)	24 (22-27)	29 (25-34)	43 (37-46)	43 (41-47)	38 (33-44)	<.0001
ALP (IU/L)	86 (71-115)	163 (116-187)	82 (63-118)	233 (122-318)	160 (118-227)	<.001
aPTT	1.1 (1.0-1.3)	1.6 (1.4-1.8)	1.0 (0.9-1.1)^*n*^^= 16^	1.0 (1.0-1.2)^*n*^^= 17^	1.1 (0.9-1.3)^*n*^^= 18^	<.0001
AST (IU/L)	1089 (443-2212)	128 (66-189)	36 (24-91)	56 (33-80)	132 (77-774)	<.0001
Creatinine (μmol/L)	94 (69-121)	76 (49-125)	67 (53-80)	68 (59-77)	62 (52-74)	<.01
Fibrinogen (g/L)	2.3 (1.9-2.7)	2.3 (1.6-3.1)^*n*^^= 13^				.96
GGT (IU/L)	96 (56-160)	149 (93-333)^*n*^^= 19^	73 (38-150)	262 (59-526)	202 (88-392)	.08
INR	1.2 (1.2-1.5)	1.7 (1.5-2.0)	1.0 (0.9-1.0)	1.0 (0.9-1.1)	1.1 (1.0-1.8)	<.0001
Neutrophil count	10 (7-13)	15 (9-22)	5 (3-6)	5 (3-7)	3 (3-4)	<.0001
Platelet count (×10^9^/L)	73 (54-120)	103 (60-198)	205 (181-297)	245 (195-308)	203 (113-241)	<.0001
Total bilirubin (μmol/L)	61 (36-83)	326 (167-521)	9 (4-12)	12 (8-20)	22 (14-127)	<.0001
WBC count (×10^9^/L)	10 (6-14)	19 (12-25)	7 (6-9)	7 (5-12)	6 (5-7)	<.0001

The results are presented as median (IQR) for continuous variables and number (%) for categorical variables of available data. Differences between groups were analyzed using the one-way analysis of variance test.

AIH, autoimmune hepatitis; ALP, alkaline phosphatase; aPTT, activated partial thromboplastin time; ASH, alcoholic steatohepatitis; AST, aspartate aminotransferase; GGT, gamma-glutamyl transferase; ICU, intensive care unit; INR, international normalized ratio; IRI, ischemia-reperfusion injury; MASH, metabolic-associated steatohepatitis; MELD, model for end-stage liver disease; PSC, primary sclerosing cholangitis; WBC, white blood cell.

a Sex and age are donor characteristics, the laboratory results are from the recipients’ post liver transplantation.

b Data from 1 patient are missing for all variables.

### 3.2 Intrahepatic fibrin(ogen) deposition

**Figure 2:**
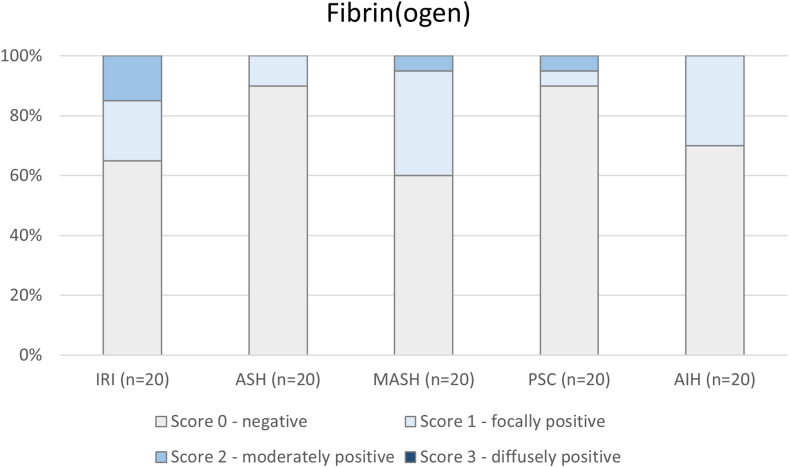
Intrahepatic fibrin(ogen) deposition is generally low in all types of liver injury. More than 60% of the liver biopsies from any liver disease were negative for fibrin(ogen) staining. Moderate positivity of fibrin(ogen) was only seen in 15% of the liver biopsies with ischemia-reperfusion injury (IRI) and 5% of the liver biopsies from patients with metabolic-associated steatohepatitis (MASH) and primary sclerosing cholangitis (PSC). AIH, autoimmune hepatitis; ASH, alcoholic steatohepatitis.

Overall, 75% of the biopsies showed no fibrin(ogen) deposition, with focal fibrin(ogen) deposition found in 20% of liver biopsies. Figure 1 shows an example of focal positive fibrin(ogen) (score 1) and moderate positive fibrin(ogen) expression (score 2). Moderate positivity of fibrin(ogen) was seen in only 3/20 (15%) of postreperfusion biopsies and in 1/20 (5%) of patients with MASH and PSC (Figure 2). Biopsies of patients with ASH and PSC were negative for fibrin(ogen) in 90% of the cases. Six (30%) biopsies of AIH patients had focal positivity for fibrin(ogen), and 14 (70%) biopsies were negative.

### 3.3 Intrahepatic platelet deposition

**Figure 3:**
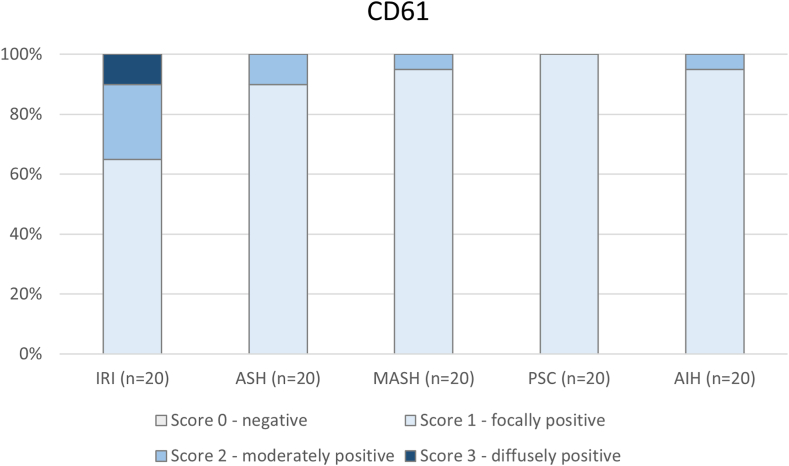
Intrahepatic platelet deposition is generally low. Intrahepatic deposition of platelets, as assessed by the platelet marker CD61, is most abundant in livers with ischemia-reperfusion injury (IRI). Most livers from patients with acute alcoholic steatohepatitis (ASH), metabolic-associated steatohepatitis (MASH), primary sclerosing cholangitis (PSC), and autoimmune hepatitis (AIH) showed scattered tiny positive granules in sinusoids.

Platelet staining was generally low in all types of injury (Figure 3). Of all cases (*N* = 100), only 2 liver biopsies with preservation injury showed many large platelet aggregates (score 3) in liver sinusoids (Figure 3). Occasional larger platelet aggregates (score 2) were most often found in preservation injury (5/20 cases; 25%). None of the PSC biopsies showed larger platelet aggregates in liver sinusoids.

### 3.4 Intrahepatic NETs

**Figure 4:**
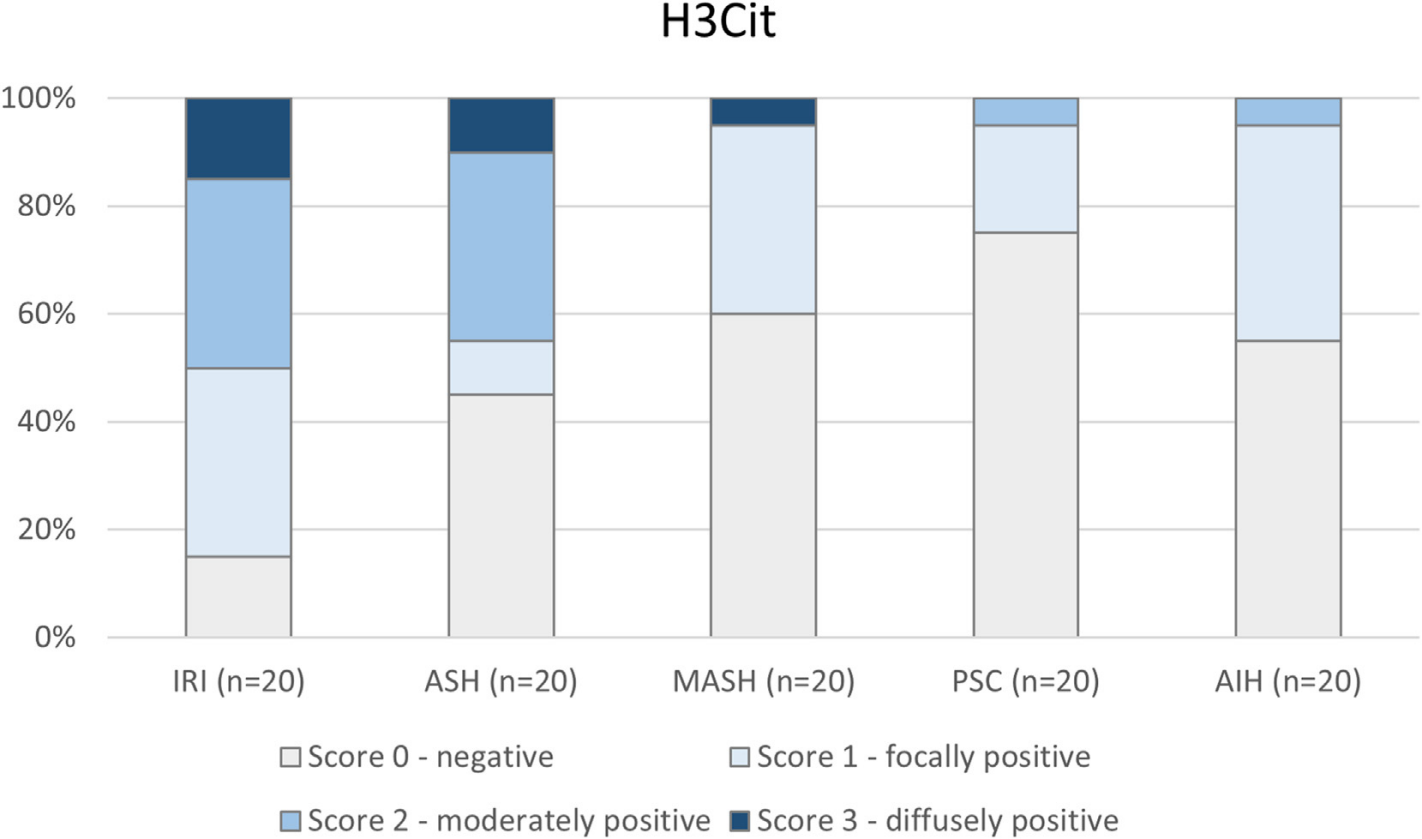
Intrahepatic neutrophil extracellular traps (NETs) in 50% of all liver biopsies. Variable expression of citrullinated histone H3 (H3Cit), a marker for NETS in donor livers with ischemia-reperfusion injury (IRI; *n* = 20), liver biopsies from patients with acute alcoholic steatohepatitis (ASH; *n* = 20), metabolic-associated steatohepatitis (MASH; *n* = 20), primary sclerosing cholangitis (PSC; *n* = 20), and autoimmune hepatitis (AIH; *n* = 20). Most NETs were found in liver biopsies with IRI and in liver biopsies from patients with acute ASH.

NET formation, as reflected by H3cit staining, was present in 85% and 50% of the liver biopsies of postreperfusion allografts and patients with ASH, respectively (Figure 4). Approximately 50% of the cases showed moderate to diffuse H3cit expression, defined as >10% of the neutrophils expressing H3cit. In contrast, less than 10% of liver biopsies from MASH, PSC, or AIH patients showed this level of H3cit expression. H3cit expression was associated with NE expression (Table 2) in livers with reperfusion injury (r = .62 [.23-.84]; *P* < .01) and ASH (r = .74 [.42-.89]; *P* < .001), suggesting a correlation between the degree of neutrophilic infiltrate and NET formation.

**Table 2:** Correlations between the degree of deposition of neutrophil extracellular traps, neutrophils, hemostatic components, and histologic injury scores for all patients and in specific patient groups.

*All patients (N = 100)*	H3Cit (0-3)	CD61 (0-3)	Fibrinogen (0-3)	NE (0-3)	VWF (0-3)
H3Cit (0-3)	-	r = .44 (.27-.59) *P* < .001	r = .16 (−.05 to .35) *P* .12	n/a	n/a
CD61 (0-3)	r = .44 (.27-.59) *P* < .001	n/a	r = .28 (.08-.46) *P* < .01	n/a	n/a
*IRI (n = 20)*					
H3Cit (0-3)	-	r = .54 (.12-.80) *P* < .05	r = .27 (−.21 to .64) *P* .25	r = .62 (.23-.84) *P* < .01	r = .55 (.12-.80) *P* < .05
NE (0-3)	r = .62 (.23-.84) *P* < .01	r = .60 (.20-.83) *P* < .01	r = .21 (−.27 to .61) *P* .37	-	r = .64 (.27-.85) *P* < .01
CD61 (0-3)	r = .54 (.12-.80) *P* < .05	-	r = .51 (.07-.78) *P* < .05	r = .60 (.20-.83) *P* < .01	r = .72 (.39-.88) *P* < .001
Histologic injury score^a^ (minimal [1]–severe [4])	r = .61 (.22-.84) *P* < .01	r = .50 (.06-.78) *P* < .05	r = .62 (.23-.84) *P* < .01	r = .51 (.08-.78) *P* < .05	r = .34 (−.14 to .68) *P* .15
*ASH (n = 20)*					
H3Cit (0-3)	-	r = .56 (.14-.81) *P* < .05	r = .28 (−.20 to .65) *P* .23	r = .74 (.42-.89) *P* < .001	r = .13 (−.34 to .55) *P* .58
NE (0-3)	r = .74 (.42-.89) *P* < .001	r = .43 (−.02 to .74) *P* .06	r = −.02 (−.47 to .44) *P* .95	-	r = .24 (−.24-.63) *P* .30
CD61 (0-3)	r = .56 (.14-.81) *P* < .05	-	r = .44 (.01-.75) *P* < .05	r = .43 (−.02 to .74) *P* .06	r = .16 (−.32 to .57) *P* .50
Histologic activity score (0-4)^b^	r = −.28 (−.65 to .20) *P* .23	r = .11 (−.36 to .54) *P* .64	r = .11 (−.36 to .54) *P* .64	r = −.19 (−.60 to .29) *P* .42	r = −.02 (−.47 to .44) *P* .94
Histologic fibrosis stage (0-4)	r = .17 (−.31 to .58) *P* .49	r = .08 (−.39 to .52) *P* .73	r = .08 (−.39 to .52) *P* .73	r = −.04 (−.48 to .42) *P* .87	r = −.38 (−.71 to .09) *P* .10
Histologic steatosis stage (0-3)	r = −.45 (−.75 to −.01) *P* < .05	r = −.18 (−.59 to .29) *P* .44	r = .02 (−.44 to .47) *P* .95	r = −.41 (−.73 to .05) *P* .07	r = .23 (−.25 to .62) *P* .33

Spearman correlation r with (95% CI).

ASH, alcoholic steatohepatitis; H3Cit, citrullinated histone H3; IRI, ischemia-reperfusion injury; n/a, not applicable; NE, neutrophil elastase; VWF, von Willebrand factor.

a Histologic injury score: 1, minimal (only focal neutrophilic infiltrate or hepatocellular necrosis); 2, mild (scattered foci of hepatocyte necrosis and neutrophilic aggregates); 3, moderate (perivenular confluent necrosis); 4, severe (bridging or pan-lobular necrosis).

b Activity is based on the sum of hepatocellular injury and lobular neutrophil scores based on the criteria from the Study of Alcohol-related LiVer Disease in Europe [19].

### 3.5 Intrahepatic NET formation and activation of coagulation

**Figure 5:**
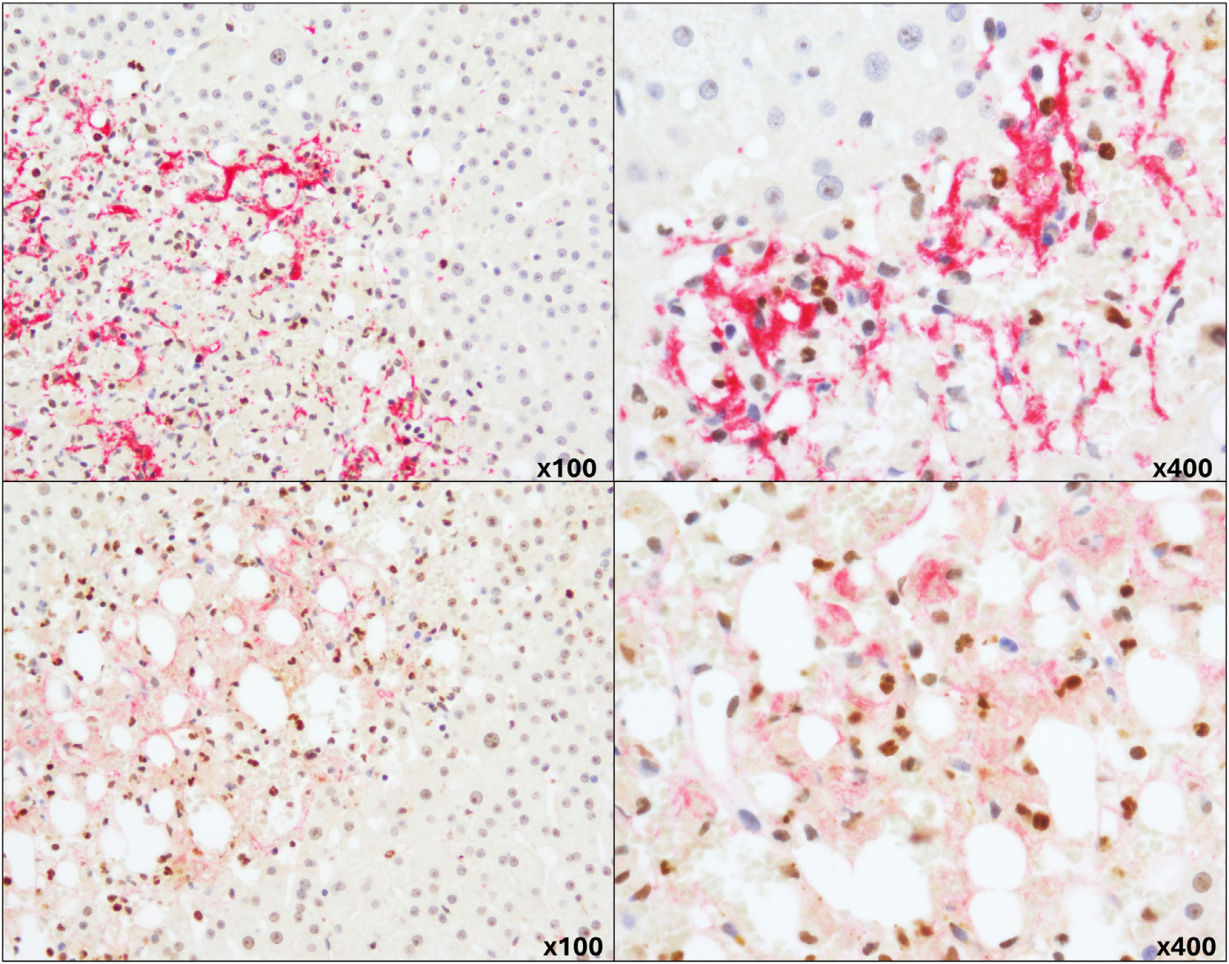
Representative images of double immunostaining for citrullinated histone H3 (brown) with CD61 (red) at the top and citrullinated histone H3 (brown) with von Willebrand factor (red) at the bottom, indicating colocalization of platelets with neutrophil extracellular traps. Magnification: left ×100; right ×400.

In the overall patient cohort, increased NET formation was associated with increased platelet but not fibrin(ogen) deposition (Table 2). As NET formation was most extensive in biopsies taken from postreperfusion allograft livers and patients with acute ASH, we performed additional staining for neutrophils, quantifying NE and VWF in these groups. In livers with reperfusion injury, NET formation was positively associated with platelet and VWF deposition (r = .54 [.12-.80]; *P* < .05 and .55 [.12-.80]; *P* < .05, respectively) but not fibrin(ogen). Double immunostaining of these liver biopsies showed colocalization of H3Cit and CD61 and H3Cit and VWF (Figure 5), suggesting an interaction between NETs and platelets in reperfusion injury. Similarly, NE staining was positively associated with platelet and VWF deposition (r = .60 [.20-.83]; *P* < .01 and r = .64 [.27-.85]; *P* < .01, respectively) but not fibrin(ogen). CD61 expression correlated positively with VWF expression (r = .72 [.39-.88]; *P* < .001) and, to a lesser extent, with fibrin(ogen) (r = .51 [.07-.78]; *P* < .05) in allograft livers with reperfusion injury.

In liver biopsies from patients with ASH, NET formation was positively associated with platelet deposition (r = .56 [.14-.81]; *P* < .05) but not with VWF or fibrin(ogen) deposition. NE positivity did not correlate with CD61, VWF, or fibrin(ogen). CD61 positivity moderately correlated with fibrin(ogen) deposition (r = .44 [.01-.75]; *P* < .05) but not with VWF deposition. Notably, all but one biopsy from patients with ASH showed VWF deposition, in contrast to allograft livers, where this was present in 11/20 (55%), *P* < .01.

### 3.6 Correlation of NET formation and hemostatic components with histologic injury scores

The degree of liver injury in postreperfusion allograft biopsies was graded following the histologic injury score from minimal to severe (1 to 4), as described above. The injury score correlated with the deposition of NETs (r = .61 [.22-.84]; *P* < .01), platelets (r = .50 [.06-.78]; *P* < .05), fibrinogen (r = .62 [.23-.84]; *P* < .01), and neutrophils (r = .51 [.08-.78]; *P* < .05) but not VWF. Liver biopsies from patients with ASH were scored on the degree of steatosis and fibrosis and activity [27]. Notably, 90% of these biopsies had the highest activity score of 4. The histologic activity score and fibrosis stage did not correlate with any of the studied components.

### 3.7 Intrahepatic NET formation and disease severity

Lastly, we correlated the expression of fibrin(ogen), CD61, and H3Cit with the severity of the disease, as reflected by laboratory measures and MELD score at the time of biopsy (Supplementary Table S2). Allograft livers were excluded from the MELD analysis. In the whole cohort, intrahepatic NET deposition moderately correlated with INR and AST (r = .24 [.04-.42]; *P* < .05), bilirubin (r = .31 [.12-.49]; *P* < .01), and MELD score (r = .29 [.07-.49]; *P* < .01) and inversely with platelet count (r = −.33 [−.5 to −.14]; *P* < .01), but fibrin(ogen) deposition did not. Platelet deposition moderately correlated with AST (r = .30 [.10-.47]; *P* < .01) and INR (r = .23 [.03-.42]; *P* < .05).

## 4 Discussion

In this study, we found no histologic evidence of intrahepatic fibrin(ogen) deposition in 75 of 100 biopsies taken from patients with different types of inflammatory liver disease. These results contrast with those from animal studies where extensive intrahepatic fibrin(ogen) deposition is often demonstrated following acute or chronic injury [6,17]. In contrast, we found histologic evidence for intrahepatic NETs in half of the samples studied, which was most pronounced in acute ASH and preservation injury and was associated with measures of disease severity.

The hypothesis that activation of coagulation contributes to the progression of liver disease by the formation of microthrombi is supported by animal studies demonstrating a decrease in both fibrin(ogen) deposition and liver injury or the development of fibrosis by prophylactic anticoagulation [6]. Here, we found low abundance fibrin(ogen) staining in only 20% of liver biopsies from 100 patients with liver disease and no deposition in 75% of the biopsies. Further, fibrin(ogen) deposition was not associated with measures of disease severity. Our findings suggest potential differences in mechanisms of liver disease progression between rodent models and humans and caution in extrapolating results from experimental studies to patients, underlining the importance of human histologic studies in understanding the pathogenesis of liver disease progression [29]. Potential explanations for the difference in fibrin(ogen) deposition in liver disease and injury in human and rodent livers could be the difference in timing. The human biopsies we studied were taken from patients suffering from liver disease for months to years, whereas animal studies usually study the progression of liver disease in days to weeks. It might be that we simply missed the fibrin(ogen) deposition phase in human liver disease and that this phase precedes clinically apparent liver disease. Moreover, intrahepatic deposition of fibrin(ogen) may proceed more efficiently in mice compared with humans, or intrahepatic fibrinolysis or fibrin-to-fibrosis conversion could take place more efficiently in humans compared with rodents. Our results are at variance with those of a study in which robust fibrin(ogen) staining was reported in liver biopsies from humans with MASH [30]. However, in this study, a polyclonal antibody was used, which may have reduced specificity compared with the monoclonal antibody we have used. Importantly, this study examined a small number (*n* = 6) of biopsies from pediatric liver disease patients. Five of 6 biopsies stained positive for fibrin(ogen). These patient characteristics were not mentioned in the published manuscript but were shared with us by the authors upon request (Drs James Luyendyk and Matthew Flick).

Intrahepatic deposition of platelets was also generally low in our study population, with only a subset of allograft livers showing moderate positive staining for CD61. Platelets have also been implicated as drivers of liver disease progression, but discrepancies between clinical observations and experimental studies have been identified [31]. Though platelets could play a direct role, the interplay between platelets and neutrophils and the generation of NETs may also be part of the explanation for why antiplatelet agents are associated with reduced progression of chronic liver disease [32,33]. Platelet-neutrophil interactions have been postulated to drive IRI in liver transplantation [34]. We found positive staining for the NET marker, H3Cit, most often in allograft IRI, which correlated with CD61 expression. Double immunostaining showed colocalization of H3Cit and CD61. It is tempting to speculate that upon hepatic reperfusion injury, activated intrahepatic platelets bind neutrophils and stimulate them to form NETs. NETs have been shown to contribute to liver injury in an animal model of IRI of the liver, and inhibition of NETs substantially decreased injury [35]. Here, we show circumstantial evidence for the role of NETs in human hepatic IRI. Further research is needed to verify their formation and pathogenic role, as there may be novel therapeutic options [36].

We also found positive staining for H3Cit in 55% of the liver biopsies from patients with ASH and 40% of biopsies from patients with MASH, though in the latter to a lower degree. The role of NETs in alcohol-associated disease is supported by both mouse models of alcohol-induced liver injury and human studies; the former has demonstrated increased NET formation in the liver with a contribution to liver damage and hepatocyte death [24,37]. In the latter, isolated neutrophils from patients with ASH show increased NET formation [24]. If NETs do form part of the pathogenesis of ASH, inhibition of NETs by therapies such as DNase might reduce its progression [38]. This might also be the case in MASH. In a mouse model of MASH fibrosis, inhibition of NETs by administration of DNase or using knockout mice resulted in decreased progression of fibrosis, with evidence of a role for activation of hepatic stellate cells by NETs [39]. In patients undergoing hepatectomy, those with MASH had elevated plasma myeloperoxidase-DNA complex levels, a marker of NETs. In a study of 17 human MASH liver biopsies, positive NET staining with immunofluorescent colocalization of NE and H3cit was found in 16 out of 17 biopsies compared with no NETs in liver biopsies from controls [26]. Importantly, the authors found NET deposition was positively associated with the histologic nonalcoholic fatty liver disease activity score and thus might represent a pathogenic mechanism contributing to disease progression. Though we did not find an association between NET deposition and the MELD score in MASH patients, we noted that the MELD scores of these patients were low.

We hypothesized that patients with PSC and AIH might show evidence of NET deposition in their livers, as neutrophils often accumulate in the liver in early autoimmune liver disease [40]. Further, the presence of NETs has been demonstrated in other autoimmune diseases, including systemic lupus erythematosus and autoimmune small vessel vasculitis [41,42], where plasma markers of NETs are associated with more severe disease. However, we did not find NETs in the livers of PSC and AIH patients, arguing against a major role of NETs in disease pathogenesis. Nonetheless, we cannot exclude a role for NETs at different disease stages or in more active disease or that NETs are formed in these patients but efficiently cleared and, therefore, not detected in biopsies.

Overall, however, H3Cit-positive staining was moderately associated with laboratory measures and scores of disease severity, which could indicate that NETs contribute to disease progression. However, one limitation of this study is the spectrum of disease severity in the different patient groups, particularly patients with acute ASH. These had a much more severe disease in comparison with the other patient groups and had a restricted range of disease severity. It is, therefore, difficult to determine whether the differences in the expression of hemostatic components we found between the different patient groups are attributed to the underlying etiology of liver disease or its severity. It could be that the increased NET and platelet deposition in acute ASH was a reflection of more severe disease and not the disease-specific mechanism of liver injury.

One of the limitations of this study is that the antibodies used are not truly specific to the studied hemostatic components. CD61, for example, is also expressed by endothelial cells and macrophages. However, we studied the CD61-positive cells that the histopathologist identified as platelets (aggregates) in hematoxylin and eosin-stained liver tissue based on the histologic appearances of the cells. In addition, interpretation of fibrin(ogen) stains in the liver is difficult as fibrin(ogen) is not only deposited in the liver from plasma but is also synthesized in hepatocytes, and this synthesis is upregulated in acute phase situations such as in inflammatory diseases, as studied here. Finally, there is an ongoing debate on how to best stain NETs in tissue. H3cit is an established marker for NETs, and although we have not performed colocalization studies of H3cit with a neutrophil marker, the correlation between H3cit and NE positivity in our samples suggests that the H3cit-positive structures are indeed NETs.

In conclusion, in contrast to results from experimental studies, we found no fibrin(ogen) deposition in 75% of liver biopsies from patients with different types of inflammatory liver disease, with only low fibrin(ogen) deposition in the other 25%. These results contrast with animal studies that found robust fibrin(ogen) deposition in animals with various types of liver injury, including fibrosis and ischemia-reperfusion injury [6,17]. We found evidence for intrahepatic NETs in half of the liver biopsies studied, principally IRI and ASH, and in the former, with close association to histologic disease activity. However, the level of NET deposition was generally low in comparison with studies in animal models, highlighting the potential difficulties of extrapolating results from animal studies to patients. Our findings suggest that NET deposition could be a pathogenic mechanism in some forms of human liver injury and disease progression, but that confirmation will require additional human histologic studies across the spectrum of disease activity and illness severity.
