# Supplementary material for: Histologic evidence of neutrophil extracellular traps and fibrin(ogen) deposition in liver biopsies from patients with inflammatory liver disease
Source: Res Pract Thromb Haemost. 2024 Dec 31;9(1):102666. doi: 10.1016/j.rpth.2024.102666 (PMC11830338; doi:10.1016/j.rpth.2024.102666)
Supplement: Supplementary material [file mmc1.docx]

**Supporting information**

**Supplementary Table S1.**

|  | **Pretreatment** | **Dilution** |
| --- | --- | --- |
| **H3Cit** | Heat in pH6 buffer (ER1; 10 min) | 1:200 |
| **CD61** | Heat in pH6 buffer (ER1; 20 min) | Pre-diluted |
| **Fibrin(ogen)** | Heat in pH6 buffer (ER1; 10 min) | 1:1000 |
| **NE** | Enzyme (E1; 15 min) | 1:1000 |
| **VWF** | Heat in pH6 buffer (ER1; 10 min) | 1:5000 |

ER1 and E1 are pretreatment options in the automated staining machine (Bond Max autostainer, Leica) we used.

**Supplementary Table S2.** Correlations between degree of deposition of neutrophil extracellular traps, neutrophils, haemostatic components and laboratory measures for all patients and in specific patient groups.

| *All patients (n=100)* | **H3Cit** (0-3) | **CD61** (0-3) | **Fibrinogen** (0-3) | **NE** (0-3) | **VWF** (0-3) |
| --- | --- | --- | --- | --- | --- |
| **MELD score** (original)†^n=80^ | r = 0.29 [0.07 – 0.49]  p < 0.01 | n.s. | n.s. | n/a | n/a |
| **AST (IU/L)** | r = 0.24 [0.04 – 0.42]  p < 0.05 | r = 0.30 [0.10 – 0.47]  p < 0.01 | n.s. | n/a | n/a |
| **INR** | r = 0.24 [0.04 – 0.42]  p < 0.05 | r = 0.23 [0.03 – 0.42]  p < 0.05 | n.s. | n/a | n/a |
| **Platelet count (*10^9^/L)** | r = -0.33 [-0.50 – -0.14]  p < 0.01 | n.s. | n.s. | n/a | n/a |
| **Total bilirubin (µmol/L)** | r = 0.31 [0.12 – 0.49]  p < 0.01 | n.s. | n.s. | n/a | n/a |
| **WBC count (*10^9^/L) / Neutrophil count** | n.s. | n.s. | n.s. | n/a | n/a |
| *IRI (n=20)* |  |  |  |  |  |
| **AST (IU/L)** | n.s. | n.s. | n.s. | n.s. | n.s. |
| **Peak AST (IU/L)** | n.s. | r = 0.55 [0.09 – 0.81]  p < 0.05 | n.s. | n.s. | n.s. |
| **Fibrinogen (g/L)** | n.s. | n.s. | n.s. | n.s. | n.s. |
| **INR** | n.s. | r = 0.52 [0.09 – 0.79]  p < 0.05 | n.s. | n.s. | n.s. |
| **Platelet count (*10^9^/L)** | n.s. | n.s. | n.s. | n.s. | n.s. |
| **Total bilirubin (µmol/L)** | n.s. | n.s. | r = 0.51 [0.07 – 0.78]  p < 0.05 | n.s. | n.s. |
| **WBC count (*10^9^/L) / Neutrophil count** | n.s. | n.s. | n.s. | n.s. | n.s. |
| *ASH (n=20)* |  |  |  |  |  |
| **MELD score** (original) | n.s. | n.s. | n.s. | n.s. | n.s. |
| **AST (IU/L)** | n.s. | n.s. | n.s. | n.s. | n.s. |
| **Fibrinogen (g/L)** | n.s. | n.s. | n.s. | n.s. | n.s. |
| **INR** | n.s. | n.s. | n.s. | n.s. | n.s. |
| **Platelet count (*10^9^/L)** | n.s. | n.s. | n.s. | n.s. | n.s. |
| **Total bilirubin (µmol/L)** | n.s. | n.s. | n.s. | n.s. | n.s. |
| **WBC count (*10^9^/L) / Neutrophil count** | n.s. | n.s. | n.s. | n.s. | n.s. |

r from Spearman correlation with [95% confidence interval]. n.s. = not significant. n/a = not applicable.

†n=80, as MELD score is not applicable for donor livers, thus these 20 biopsies are excluded from this analyses.

Abbreviations: H3Cit, citrullinated histone 3; NE, neutrophil elastase; VWF, von Willebrand factor; MELD, model for end-stage liver disease; AST, aspartate aminotransferase; INR, international normalized ratio**;** WBC, white blood cell, IRI, ischemia-reperfusion injury; ASH, alcohol-associated steatohepatitis.


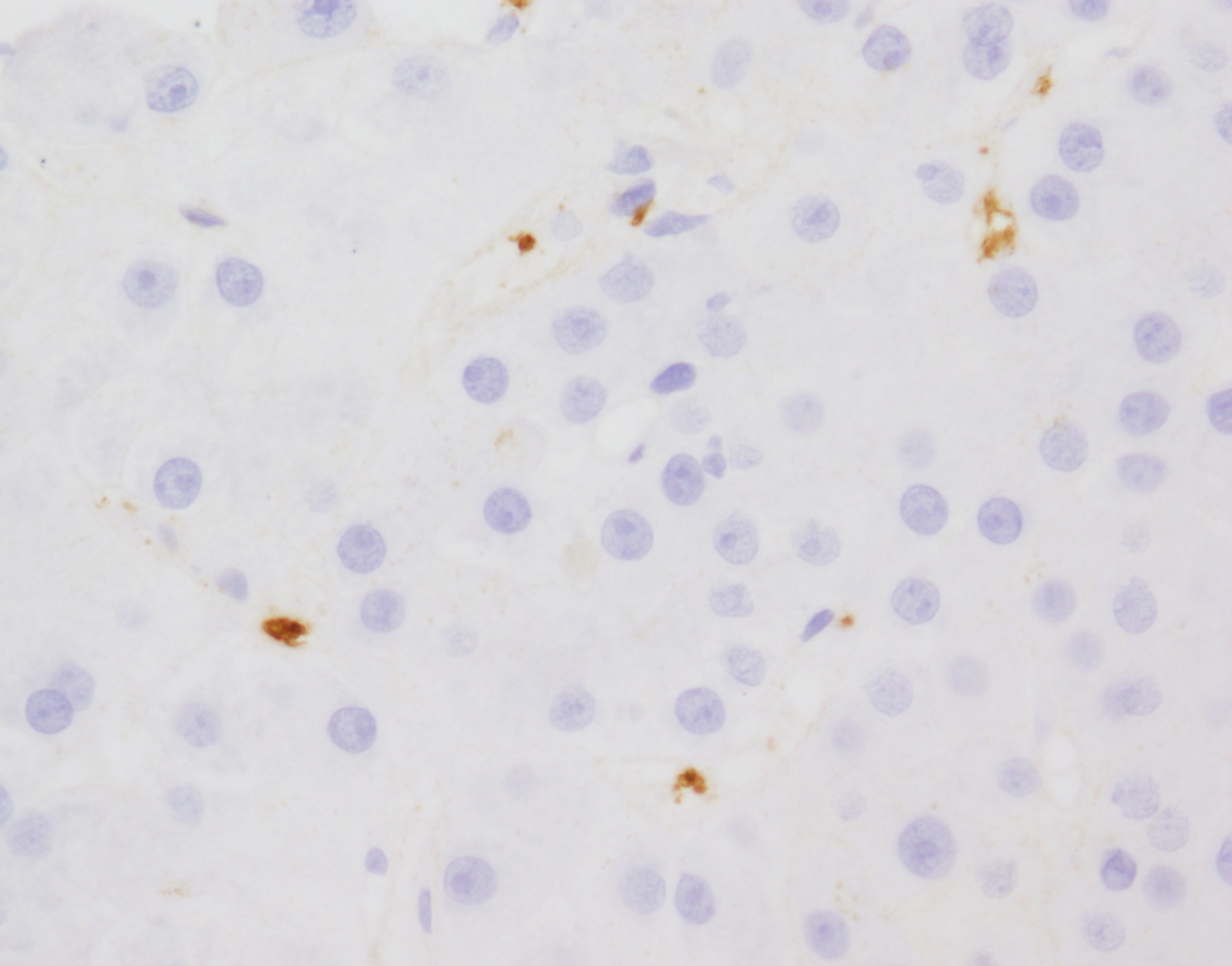


**Supplementary Figure.** Representative high magnification picture of CD61 staining showing small granular staining inside sinusoids indicative for platelets.
